# Primary Carcinoid Tumor of the Ileal Efferent Limb of an Ileovesicostomy: A Case Report

**DOI:** 10.1155/2011/191702

**Published:** 2011-10-26

**Authors:** Adamantios M. Mellis, Daniel C. Parker, David D. Buethe, Gennady Slobodov

**Affiliations:** Department of Urology, University of Oklahoma Health Sciences Center, Oklahoma City, OK 73104, USA

## Abstract

We report on the evaluation and management of a 47-year-old white male found to have primary carcinoid tumor of the ileal segment of his diverting ileovesicostomy thirty-five months after initial creation. Subsequent to presentation with intermittent gross hematuria, CT urogram highlights an 8 mm enhancing lesion near the enterovesical junction of urinary diversion. Office cystoscopy confirms presence of a lesion that was later endoscopically resected and found to be a well-differentiated carcinoid tumor. Evaluation with serum markers, direct visualization utilizing endoscopy, and imaging was without finding of alternate primary or metastatic lesions. The patient ultimately had the proximal ileal portion of his ileovesicostomy excised and the distal portion converted into an ileal conduit. After briefly discussing the carcinoid tumor and the carcinoid syndrome it may cause, we review the literature on the incidence of carcinoid tumors in a population requiring the use of intestine in the urinary tract.

## 1. Introduction

Carcinoid tumors were first described over a century ago by Lubarsch with the term *karzinoide* used by Oberndorfer in 1907 to describe tumors that were slower growing than typical adenocarcinomas [1]. Carcinoid is a slow-growing tumor originating from cells of the neuroendocrine system. Carcinoid is most often found in the gastrointestinal system, namely, in the ileum and the appendix. However, carcinoid also has a high incidence in the lung. The incidence of carcinoid tumors ranges from 2.5 to 5 per 100,000 [2]. Carcinoid has the potential to become malignant and cause the carcinoid syndrome, resulting from excessive activation of biogenic amines, bradykinins, and tachykinins throughout systemic circulation. The carcinoid syndrome causes a plethora of symptoms including watery diarrhea, wheezing, abdominal pain, and heart failure. Carcinoid of the small bowel may account for 90% of the incidence of carcinoid syndrome [3].

## 2. Case Report

**Figure 1:**
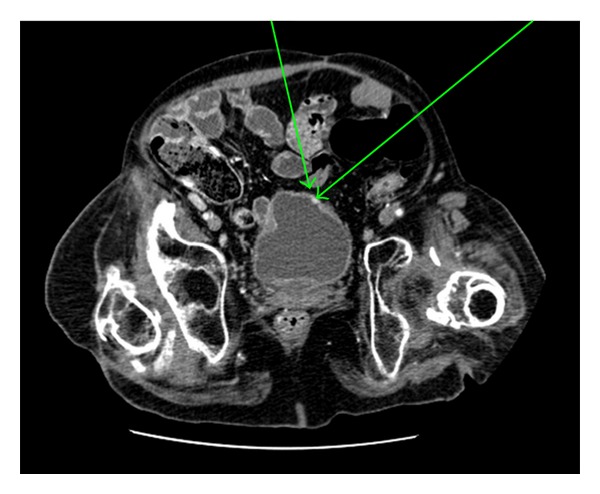
An arterial phase view of the carcinoid tumor seen on CT urogram. The tumor is located at the anterior portion of the cystoplasty along the bladder-ileum border (arrows pointing to area of concern for tumor).

**Figure 2:**
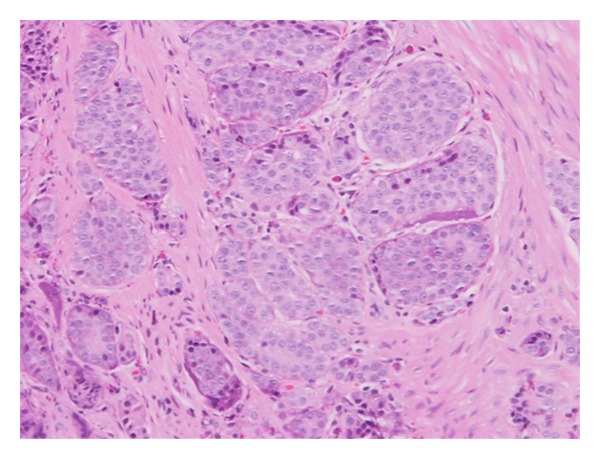
A high-powered histologic view of the carcinoid tumor. This tumor demonstrates the carcinoid tumor cell's characteristic ovoid nuclei with scant cytoplasm along with a fibrovascular stroma.

A 47-year-old man, with paraplegia related to a T7 spinal cord injury suffered at the time of a motor vehicle collision nearly 30 years prior, originally presented carrying the diagnosis of neurogenic bladder that had been managed by indwelling foley catheter. At that time, physical exam demonstrated the manifestations of urethral erosion which would later be complicated by development of a necrotic phallus and eventual loss of this appendage by means of partial penectomy. As a means of urinary diversion, he underwent creation of an ileovesicostomy that was later revised due to redundancy of the efferent limb. At the time of revision, a concurrent bladder neck ligation was performed due to persistent urinary incontinence per urethra. Thirty-five-month status after original diverting procedure, a CT urogram was obtained to evaluate complaint of intermittent gross hematuria. This study demonstrated an enhancing 8 mm mass near the enterovesical anastamosis of the ileovesicostomy (Figure 1). Office cystoscopy confirmed the finding, and subsequent cold-cup biopsy was obtained using a flexible cystoscopy in the operative suite. An acquired pathologic specimen was consistent with well-differentiated neuroendocrine carcinoma, consistent with carcinoid (Figure 2). The lesion was confined to the ileal mucosa. The four biopsy sites measured approximately 2 mm each in greatest diameter. Special histologic markers for carcinoid, including synaptophysin, chromogranin, and pankeratin, stained positive. An ensuing octreotide scan, esophagogastroduodenoscopy, and colonoscopy were all without evidence of alternate primary or metastatic site. Given the paucity of literature regarding such a lesion, no clearly defined algorithm outlined on how to proceed. Thus, repeat cold-cup biopsy was performed at site of previous excision. Pathologic tissue was without evidence of malignancy. Despite negative repeat biopsy, the patient elected to proceed with excision of the proximal aspect of his ileovesicostomy with the distal portion converted into an ileal conduit.

## 3. Discussion

According to the 2010 National Comprehensive Cancer Network (NCCN) guidelines for neuroendocrine tumors, triple phase CAT scan or MRI of the abdomen or pelvis is the first-line imaging modality for evaluation of a carcinoid tumor [4]. Octreotide scan, colonoscopy, and small bowel imaging may be considered as appropriate. 

Treatment of a localized carcinoid in the ileum should involve resection of the affected segment of ileum with associated mesentery. For patients with the carcinoid syndrome, octreotide is considered first line therapy. Depending on the organ system, therapy for metastatic carcinoid may differ. Treatment may include chemotherapy and, if feasible, resection of metastases. Precise treatment for each organ system affected by carcinoid syndrome is beyond the scope of this review. 

To our knowledge, this is the first case of a carcinoid tumor within an ileovesicostomy described in the urologic literature. A MEDLINE review of “carcinoid tumors” and “urinary diversion” reveals five results, a testament to the rarity of these tumors and among the reports of carcinoid in intestine used in other forms of the urinary tract; there are three cases seen in an ileal conduit [5–7] and one case of carcinoid in an ileal neobladder [8]. The fifth case included in the MEDLINE review is an appendiceal carcinoid tumor with metastasis to the small and large bowels and omentum causing urinary obstruction and the carcinoid syndrome. The authors of that paper created a cutaneous ureterostomy to relieve the obstruction [9]. 

Among the cases involving intestine used in urinary diversion, Frese et al. reported on the case of carcinoid in an ileal neobladder [8]. Their patient is a 64-year-old man who underwent radical cystectomy and creation of the neobladder for invasive transitional cell carcinoma. The patient's lesion was discovered endoscopically upon routine surveillance cystoscopy. Klink et al. presented the case of a 74-year-old woman with gross hematuria from her ileal conduit and resulting tumor that was carcinoid. This patient's conduit was ultimately excised with the creation of a new conduit [5]. Kerfoot et al. report a 61-year-old woman with carcinoid of an ileal conduit that leads to conduit stenosis and hydronephrosis [6]. Finally, Kochevar presents the case of a 55-year-old woman with abdominal pain, urinary obstruction, and recurrent urinary tract infections. Their patient had a stenosis of her ileal conduit as a result of carcinoid [7]. The area of concern in the conduit was resected, and an end-to-end anastomosis was created. In all four cases the carcinoid syndrome was not present as summarized in Table 1.

We report a case of a unique occurrence, carcinoid in intestine used in the urinary tract. More specifically, we show the first case reported in the urologic literature of a carcinoid tumor in a segment of ileum used in a bladder augment. Due to the low incidence of this unique type of tumor in an urologic patient, definitive treatment algorithms are absent. Interestingly, this patient had a CT scan prior to his initial surgery of bladder augment with ileovesicostomy that was free of tumor burden. The question of whether the use of this segment of ileum in bladder augmentation contributed to the patient's carcinoid tumor is an interesting one. However, due to the low incidence of this tumor type, it is not possible to draw conclusions about the cause of the carcinoid tumor at this time.

## Figures and Tables

**Table 1:** Summary showing the initial presentation, diagnostic studies, and treatment of carcinoid in urinary diversions.

Author	Duration of urinary diversion	Reason for diversion	Symptoms	Diagnostic studies	Treatment
Frese et al.	~9 years	Invasive TCC of the bladder	None (found during surveillance cystoscopy)	Cystoscopy	Urethrectomy, removal of neobladder, creation of ileal conduit
Kerfoot et al.	9 years	Invasive adenocarcinoma of the bladder	Intermittent bilateral flank pain	Intravenous pyelogram (IVP)	Excision of obstruction with end-to-end reanastomosis
Klink et al.	28 years	Pelvic exenteration for cervical cancer	Gross hematuria	CT abdomen/pelvis, IVP, cystoscopy, urine cytology	Resection of the ileal conduit and creation of a new one
Kochevar	12 years	Recurrent pyelonephritis secondary to ureteral stenosis	Abdominal Pain, recurrent UTIs	Ultrasonography, IVP, cystoscopy	Excision of obstruction with end-to-end reanastomosis
